# Validation of the Indonesian Brief Screening Instrument for Epilepsy

**DOI:** 10.7759/cureus.115371

**Published:** 2026-08-28

**Authors:** Fitri Octaviana, Theodore A Nathan, Adrian R Harsono, Winnugroho Wiratman, Luh A Indrawati, Manfaluthy Hakim, Ahmad Y Safri, Nurul Fadli, Astri Budikayanti

**Affiliations:** 1 Neurology, Dr. Cipto Mangunkusumo National Central General Hospital, Jakarta, IDN; 2 Neurology, Faculty of Medicine, Universitas Indonesia, Jakarta, IDN; 3 Neurology, Rumah Sakit Universitas Indonesia, Depok, IDN; 4 Neurology, Dr. Cipto Mangunkusumo National General Hospital, Jakarta, IDN

**Keywords:** cross-cultural adaptation, epilepsy, indonesia, questionnaires, screening, surveys, validation studies

## Abstract

Background

Epidemiological studies on epilepsy in Indonesia are scarce, and population-based studies are non-existent. Additionally, no screening questionnaires for epilepsy are available in Indonesia, making future large-scale studies difficult. Therefore, we aimed to validate a short Indonesian screening instrument for epilepsy.

Methods

An English-language brief screening instrument for the ascertainment of epilepsy was translated into Indonesian through cross-cultural adaptation. The translated questionnaire was distributed to 125 subjects with epilepsy at the neurology clinic in Dr. Cipto Mangunkusumo National Central General Hospital and 125 subjects with no known history of epilepsy for validation.

Results

The translated Indonesian Brief Screening Instrument for Epilepsy demonstrated good reliability, with an overall Cronbach’s alpha of 0.856. A total of 125 cases and 125 controls were analyzed. The sensitivity and specificity of the Indonesian Brief Screening Instrument for Epilepsy varied based on definitions, ranging from 97.6% to 100% and 60.8% to 97.6%, respectively. Under the broadest screen definition, the Indonesian Brief Screening Instrument for Epilepsy achieved 100% sensitivity (95% CI: 97.09-100) and 60.8% specificity (95% CI: 51.67-69.41), with a positive predictive value (PPV) of 1.77% (95% CI: 1.42-2.19) and a negative predictive value (NPV) of 100% (95% CI: 95.26-100). The tool’s high NPV indicates strong ability to rule out epilepsy in screen-negative individuals.

Conclusion

The Indonesian Brief Screening Instrument for Epilepsy is a valid and reliable screening instrument for epidemiological studies in Indonesia.

## Introduction

Epilepsy is a significant public health concern worldwide, with an estimated prevalence of approximately one percent of the global population [1]. In Indonesia, current estimates suggest that the prevalence of epilepsy ranges between 4.3 and 8 per 1,000 people. However, these estimates are derived predominantly from hospital-based studies conducted in single centers, which inherently introduce a selection bias [2-4]. Consequently, the true prevalence of epilepsy in Indonesia remains uncertain.

To accurately determine the prevalence of epilepsy across Indonesia, a multi-centered, population-based study would be ideal. Such a study would capture a more representative sample of the diverse populations within Indonesia’s archipelago. However, conducting such extensive research requires an efficient and reliable screening tool that is brief yet sensitive enough to identify epilepsy cases accurately within a population.

Currently, no validated brief screening questionnaire specifically designed for epilepsy exists for the adult population in Indonesia. While various screening tools for epilepsy have been developed and tested globally, their performance differs across populations. Among the existing screening tools, the brief screening questionnaire for epilepsy developed by Ottman et al. in 2010 stands out as a widely utilized instrument that is specifically designed to screen for epilepsy, and has been translated to several languages including Bahasa Melayu [5-8]. This questionnaire comprises nine items that assess febrile seizures, seizure disorders, various seizure characteristics, and seizure mimics. It has demonstrated excellent sensitivity and specificity in detecting epilepsy cases across diverse populations. Its robust performance as a screening tool has led to its translation and validation in various countries, confirming its adaptability to different cultural and linguistic contexts [6-8]. This study aimed to translate and culturally adapt the Brief Screening Instrument for Epilepsy into Bahasa Indonesia and to evaluate its internal consistency and diagnostic accuracy, including sensitivity and specificity, in a tertiary-care case-control population. This initial validation was intended to provide evidence for the instrument's potential use in future epidemiological research; its performance in community-based populations requires further evaluation.

## Materials and methods

This study is a case-control validation study conducted at Dr. Cipto Mangunkusumo National Central General Hospital (CMNCGH). This study aims to validate the Brief Screening Instrument for Epilepsy, which has been translated and adapted into Bahasa Indonesia, originally validated by Ottman et al. [5]. Ethical approval was obtained from the Medical Research Ethics Committee of the Faculty of Medicine, Universitas Indonesia-Dr. Cipto Mangunkusumo National Central General Hospital, Jakarta, Indonesia (KET-1238/UN2.F1/ETIK/PPM.00.02/2024, dated: August 30, 2024). Informed consent was obtained from all participants prior to participation.

Selection of screening instrument

The brief epilepsy screening instrument originally validated by Ottman et al. was selected for adaptation and translation into Bahasa Indonesia [5]. The questionnaire comprised nine items divided into three sections: Section 1 (Q1) assessed the occurrence of febrile-related seizures, Section 2 (Q2) addressed non-febrile seizure disorders, including epilepsy, and Section 3 which consisted of Items 3A to 3G (Q3A-Q3G) contained a breakdown of different seizure mimics and characteristics, in response to a negative answer to Q2. This tool demonstrated excellent performance in its original form, with a sensitivity of 96%, and specificity of 93% for epilepsy [5].

To ensure cultural and linguistic appropriateness, the translation process adhered to the rigorous three-stage cross-cultural adaptation framework established by the International Quality of Life Assessment (IQOLA) project [9]. Stage 1 involved evaluating and refining the conceptual equivalence and cultural suitability of the Indonesian version. Stage 2 focused on empirical testing to confirm the validity of the assumptions underlying the instrument’s construction and scoring. Finally, stage 3 assessed the psychometric properties of the translated questionnaire to establish its validity and reliability within the Indonesian context.

Translation and Cross-Cultural Adaptation

The translation and adaptation process ensured the instrument's conceptual equivalence and cultural relevance for the Indonesian population. The process began with forward translation, where two independent board-licensed Indonesian translators translated the original English questionnaire into Bahasa Indonesia. These two versions were then compared and consolidated into a single reconciled version after resolving discrepancies.

Next, a back translation was performed by bilingual experts who were later involved in the expert panel review and as authors in this study (FO, AB, ARH). These individuals had not participated in the initial forward translation. While this approach deviates from the conventional use of independent back translators, it was deemed appropriate given the substantial differences in epilepsy-related terminology between English and Bahasa Indonesia. Certain expressions in the original questionnaire lack direct equivalents or may carry different connotations in Bahasa Indonesia, requiring contextual interpretation by experts familiar with both languages and epilepsy-related concepts. The reconciled Indonesian version was translated back into English to verify its accuracy and fidelity to the original content.

Finally, an expert panel review was conducted. The panel comprised three individuals proficient in both English and Bahasa Indonesia (FO, AB, ARH), each with prior experience in cultural adaptation of clinical tools. All experts are neurologists with expertise in epilepsy, two of which were epileptologists. The panel then carefully compared the back-translated version with the original English version. Any differences were discussed and resolved to ensure the final version retained the intended meaning and was culturally appropriate.

Cognitive Debriefing and Finalization

To try out the translated questionnaire, 10 Indonesian-speaking participants were interviewed to evaluate their understanding of the items and to identify any difficulties they experienced while responding. Feedback from this process was used to refine the questionnaire, ensuring clarity and ease of use. From the cognitive debriefing process, we received some feedback for Items 3B, 3C, and 3G. A second expert panel review was conducted in response to this feedback, involving the same experts from the initial translation and adaptation process. Based on their consensus, final adjustments were made while preserving the tool’s content and face validity. The questionnaire was then approved for subsequent validation testing. The changes made are listed below.

In Item 3B, a subject answered “yes/possible” to “pincang” (“going limp”) due to having a limp or weakness in their right foot. The term was then changed to “pincang tanpa sebab” (“going limp without clear reason”) to avoid this association.

In Item 3C, a subject answered “yes/possible” due to “tatapan kosong” (“spacing out”) based on experiences of zoning out or day-dreaming when feeling stressed. The term was then changed to “tatapan kosong yang tidak dapat anda kendalikan” (“uncontrollable blank stare”) to clarify that the episodes are involuntary.

Finally, in Item 3G, a subject needed further clarification for the terms “kejadian tidak biasa lainnya yang berulang” (“other type of repeated unusual spells”). The terms were changed to “kejadian tidak biasa lainnya yang berulang, dan tidak dapat dijelaskan” (“repeated unusual spells and cannot be explained”) to improve clarity and brought the meaning closer to that of the original English item.

Translation Equivalence and Acceptability

The translated questionnaire was equivalent to the original English version, with the only major adjustment made to Item 3G to accommodate cultural and linguistic differences. The questionnaire consisted of nine items divided into three sections. Item 1 (Q1) addressed the occurrence of febrile-related seizures, Item 2 (Q2) inquired about non-febrile seizure disorders, including epilepsy, and Items 3A to 3G (Q3A-Q3G) explored different seizure mimics and characteristics. Item 3A explored alternative terms commonly used to describe seizures, while Item 3B focused on experiences of uncontrolled body movements. Item 3C addressed changes in mental state, and Item 3D examined episodes of daydreaming. Item 3E investigated sensitivity or unusual reactions to light, whereas Item 3F inquired about jerking or clumsiness upon awakening. Finally, Item 3G covered the occurrence of other unusual or unexplained spells. For Item 3A, the terms “seizure, convulsion, fit, or spell” were replaced with four Indonesian words, “kejang,” “ayan,” “step,” and “kerasukan,” to reflect culturally relevant terminology. In Item 3B, the phrase “saat aktivitas atau saat bangun tidur” (during waking hours) was added after “jerking” to clarify and exclude physiological hypnic jerks. For Item 3F, the term “secara tidak sengaja” (accidentally) was added after “flying from your hands” to emphasize unintentional flinging rather than purposeful actions. In Item 3G, the word “spells” was rephrased as “kejadian tidak biasa yang berulang” (repeated unusual events). The comparison between the original and Indonesian version of Brief Screening Instrument for Epilepsy (IBSIE) questionnaire after several changing and adjustments were presented in Table 1.

**Table 1:** Items of the translated questionnaire in comparison to the original The brief epilepsy screening instrument originally validated by Ottman et al. was selected for adaptation and translation into Bahasa Indonesia, after receiving permission from Dr. Ottman [5].

Item	Original	Brief Screening Instrument of Epilepsy in Bahasa Indonesia
Q1: Febrile seizures	Did anyone ever tell you that you had a seizure or convulsion caused by a high fever when you were a child?	Apakah ada yang pernah memberitahu Anda bahwa Anda mengalami kejang, atau ayan yang disebabkan oleh demam tinggi ketika Anda masih anak-anak?
Q2: Epilepsy/Seizure disorders	[Other than the seizure[s] you had because of a high fever] Have you ever had, or has anyone ever told you that you had, a seizure disorder or epilepsy?	[Selain dari kejang yang Anda alami karena demam tinggi] Apakah Anda pernah mengalami, atau ada yang pernah memberitahu Anda bahwa Anda mengalami kejang atau epilepsi?
Q3: Seizure mimics/characteristics		
Q3A: Other terms for seizure	A seizure, convulsion, fit or spell under any circumstances?	Kejang, ayan, step atau kerasukan dalam kondisi apa pun?
Q3B: Uncontrolled body movements	Uncontrolled movements of part or all of your body such as twitching, jerking, shaking or going limp?	Gerakan yang tidak terkendali dari sebagian atau seluruh tubuh Anda, seperti kedutan, sentakan (saat aktivitas atau saat bangun tidur), gemetar, atau pincang tanpa sebab?
Q3C: Changes in mental state	An unexplained change in your mental state or level of awareness; or an episode of “spacing out” that you could not control?	Perubahan mental atau tingkat kesadaran yang tidak dapat dijelaskan, atau tatapan kosong tanpa sebab, yang tidak dapat Anda kendalikan?
Q3D: Daydreaming	Did anyone ever tell you that when you were a small child, you would daydream or stare into space more than other children?	Apakah ada yang pernah memberitahu Anda bahwa ketika Anda masih anak-anak, Anda lebih sering melamun atau menatap kosong dibandingkan anak-anak lain?
Q3E: Reactions to light	Have you ever noticed any unusual body movements or feelings when exposed to strobe lights, video games, flickering lights, or sun glare?	Apakah Anda pernah memperhatikan adanya gerakan tubuh (misal: sentakan, gerakan tubuh tidak terkendali) atau perasaan yang tidak biasa saat terpapar sorotan cahaya, video game, lampu berkedip-kedip, atau silau matahari?
Q3F: Jerking/clumsiness upon awakening	Shortly after waking up, either in the morning or after a nap, have you ever noticed uncontrollable jerking or clumsiness, such as dropping things or things suddenly “flying” from your hands?	Sesaat setelah bangun tidur, baik di pagi hari atau setelah tidur siang, apakah Anda pernah memperhatikan adanya sentakan yang tidak terkendali atau gerakan ceroboh, seperti menjatuhkan benda, atau benda tiba-tiba "terlempar" dari tangan Anda secara tidak sengaja?
Q3G: Other unusual spells	Have you ever had any other type of repeated unusual spells?	Apakah Anda pernah mengalami kejadian tidak biasa lainnya yang berulang, dan tidak dapat dijelaskan?

Operational Definition

Responses to each question in the screening instrument were classified as positive if the participant answered “yes” or “possible.” Four criteria were used to define a positive screen, consisting of a positive response to: (A) any question in the screening instrument (any positive), (B) any question from Q2 to Q3G (any positive excluding febrile seizures), (C) either Q2 or Q3A (any seizures), and (D) Q2 only, indicating epilepsy specifically (Table 1).

Pilot Study

An initial pilot study was conducted on 30 subjects, consisting of 15 cases (individuals with a known history of epilepsy, confirmed through clinical evaluation and EEG by an epileptologist) and 15 controls (individuals from the general population without a history of epilepsy), using consecutive sampling. Cases were enrolled at the epilepsy outpatient clinic in Dr. Cipto Mangunkusumo National Central General Hospital (CMNCGH) in Jakarta, Indonesia, while controls were patients, or family and friends of patients visiting other clinics at CMH besides the neurology clinic. Inclusion criteria for the case group were Indonesian-speaking adults aged 18 years or older who had been diagnosed with epilepsy for over a month. Inclusion criteria for the control group were Indonesian-speaking adults aged 18 years or older without a known history of epilepsy. Subjects with intellectual disability or cognitive decline were excluded from this study. Analysis was done using Cronbach’s alpha to test the reliability of the translated questionnaire. Once the questionnaire was deemed reliable, it would then proceed to be distributed on a much larger sample size to undergo repeated testing for reliability, followed by validation [10].

Field Study

A sample size of 180 subjects was determined to be adequate for validation based on previous literature, which recommends 20 participants per questionnaire item [11]. However, a more recent study suggested that for patient-related questionnaires, a robust validation sample should include at least 250 participants [12]. Accordingly, we administered the questionnaire to 250 participants: 125 cases and 125 controls, using consecutive sampling.

All questionnaires were administered through face-to-face interviews by a general practitioner (GP) who received training from a neurologist and also the main author (FO) in standardized administration of the questionnaire. The interviewer conducted supervised interviews under neurologist supervision. Because only one interviewer administered the questionnaire, inter-rater agreement was not assessed. Informed consent was obtained prior to participation, and demographic data (age and gender) and clinical characteristics were also collected.

Cases were Indonesian-speaking adults aged 18 years old or older who had been diagnosed with epilepsy for over one month according to International League Against Epilepsy (ILAE) diagnostic criteria. The diagnosis was established by neurologists, and all case participants underwent EEG evaluation. They were recruited at the epilepsy outpatient clinic at CMH in Jakarta, Indonesia. Controls consisted of other patients and family members or friends of patients visiting outpatient clinics other than the neurology and epilepsy clinics. The same inclusion and exclusion criteria for the case and control groups were used in both the pilot and field studies.

Diagnostic Interview

Participants in the control group who were screen-positive were invited to provide informed consent for a secondary diagnostic interview. The structured interviews were conducted by neurologists who also the authors (FO, AB, ARH) in distinguishing epileptic from non-epileptic events. Questions were adapted from Beniczky et al. as presented in Table 2 [13].

**Table 2:** Questions used in the structured diagnostic interview for screen-positive individual Questions adapted from Beniczky et al. [13], with permission.

Parts/Section	Questions
1. History of Seizure	Have you ever experienced convulsions, temporary episodes of altered consciousness/awareness, altered responsiveness, or altered ability to speak?
	Have you ever experienced temporary episodes of strange behavior or involuntary movements/jerks?
2. Onset, Timing & Frequency of Seizure	At what age did the episode/seizure first occur?
	Were you having a fever at the time when the episode/seizure occurred?
	How many times have you experienced these episodes/seizures?
	How many times did you experience these episodes/seizures over the last month/year?
	Do you usually experience these episodes/seizures while awake, during sleeping, or both?
	Did these episodes/seizures come about immediately/soon after:
	- Waking up?
	- Urinating or defecating?
3. Characteristic of Seizure	What happens during these episodes/seizures? Did you experience:
	- Lip smacking or chewing?
	- Staring with impaired awareness?
	- Sudden irregular jerks? in which limb(s)?
	- Stiffening or shaking of the limbs/body?
	- Sudden loss of consciousness with a slump?
	Were you conscious during these episodes/seizures?
	How long was each episode/seizure?
	Do you know/has someone noticed whether your eyes were open or closed during the episode/seizure?
4. Pre-Ictal	What happens before these episodes/seizures? Did you experience:
	- Headaches?
	- A change in skin color/turning pale?
	- Nausea?
	- Feeling faint or lightheaded?
	- Excessive sweating?
5. Neuroimaging	Have you ever had a brain CT scan or MRI?
	(If subject(s) answers “yes” the question) What did the doctor explain regarding the brain CT scan or MRI? Was any lesion/damage/tumor/malformation/infection(s) found in the CT scan or MRI?

Participants in the case group did not require this diagnostic interview as they have been diagnosed with epilepsy. The interview required data such as current age, age at first afebrile seizure, and details on seizure characteristics, seizure mimics, and neuroimaging. Previous multi-center studies have demonstrated its reliability to classify epilepsy types with an agreement rate of 83.2% and a coefficient of 0.82, indicating near-perfect concordance with expert classification [14]. Through the diagnostic interview, a participant classified as "non-epileptic" were considered a false-positive screen and all other participants were deemed true positives. Participants diagnosed with epilepsy who were not under medical follow-up were advised to seek evaluation and management at the neurology clinic.

Statistical analysis

After completing the questionnaires, the responses were compiled and analysed using SPSS version 31.0 (IBM Corp., Armonk, NY, USA). The distribution of continuous variables was assessed using the Kolmogorov-Smirnov test. Because the continuous data were not normally distributed, they were summarized using median and range and compared between groups using the Mann-Whitney U test. Categorical variables were presented as frequencies and percentages and compared using the Chi-square test. Cronbach’s alpha was calculated to assessed internal consistency. All questionnaire items were coded in the same direction: "yes" or "possible" responses were classified as positive, whereas "no" or "don't know" responses were classified as negative; therefore, reverse coding was not required. Sensitivity, specificity, positive predictive value (PPV), and negative predictive value (NPV) were calculated for each operational definition. Sensitivity was defined as the proportion of epilepsy cases who screened positive and specificity as the proportion of controls who screened negative. Sensitivity and specificity were reported with 95% confidence intervals calculated using the Clopper-Pearson exact binomial method. Because the case-control design fixes the proportion of cases and controls and therefore does not reflect population prevalence, PPV and NPV were not calculated directly from the study sample. Instead, they were model derived using Bayes' theorem at an assumed epilepsy prevalence of 0.7%, consistent with rates reported in socio-economically comparable Southeast Asian populations [15]. Their 95% confidence intervals were obtained using the standard-logit method of Mercaldo et al. [16], which propagates the sampling uncertainty of sensitivity and specificity while holding the assumed prevalence fixed. These predictive values should therefore be interpreted as prevalence-dependent model estimates rather than directly observed predictive values.

## Results

The median age for the case group was 33 years (18-86), with a slightly higher proportion of females (52%). The majority were not married (57.6%), and the most common level of education attained was senior high school (43.2%). The median age of epilepsy onset was 19 years (1-74), with focal seizures being the dominant seizure type (84.8%). Most cases were on polytherapy (56%). The median age for the control group was 36 years (18-71), with a higher proportion of males (60%). The majority were married (68%), and most had completed senior high school (66.4%). There were no significant differences in age or sex between the case and control groups. However, marital status and educational level differed significantly (p < 0.001). No missing data were identified. All demographic and clinical characteristics of our subjects are presented in Table 3.

**Table 3:** Demographic and clinical characteristics of subjects (n=250) †Mann-Whitney U; *Chi-square; **Likelihood ratio. ASM: Anti-Seizure Medication.

Variables	Cases (n=125)	Controls (n=125)	P value
	N (%)	N (%)	
Age (median (min-max))	33 (18-86)	36 (18-71)	0.056†
Sex			0.057*
Male	60 (48)	75 (60)	
Female	65 (52)	50 (40)	
Marital status			<0.001*
Not married	72 (57.6)	40 (32)	
Married	53 (42.4)	85 (68)	
Educational level			<0.001**
No formal education	6 (4.8)	0 (0)	
Elementary	7 (5.6)	4 (3.2)	
Junior High	21 (16.8)	5 (4)	
Senior High	54 (43.2)	83 (66.4)	
Undergraduate/Postgraduate	37 (29.6)	33 (26.4)	
Age of Epilepsy Onset (years) (median (min-max))	19 (1-74)	-	-
Seizure type		-	-
Focal	106 (84.8)		
General	19 (15.2)		
Number of ASMs used		-	-
Monotherapy	55 (44.0)		
Polytherapy	70 (56.0)		

Reliability

The internal consistency of the translated questionnaire was assessed using Cronbach's alpha based on 30 participants in the pilot study. The overall Cronbach's alpha for the nine-item scale was 0.857, indicating good reliability. An item-total statistics analysis revealed that most items had corrected item-total correlations above the commonly accepted threshold of 0.3, with the exception of Item 1 (corrected item-total correlation = -0.057). The removal of Item 1 would result in a slight increase in Cronbach's alpha to 0.882. A repeated analysis was conducted on 250 participants (Table 4) which yielded an overall Cronbach's alpha of 0.856, maintaining good reliability. Similarly, item-total statistics analysis revealed that only Item 1 had a corrected item-total correlations below the commonly accepted threshold of 0.3 (0.229).

**Table 4:** Reliability and item-total correlations (n=250)

Reliability Statistics			
	N of items	Cronbach’s Alpha based on Standardized items	Cronbach’s Alpha
	9	0.838	0.856
Item-Total Statistics			
	Scale means if item deleted	Corrected item-total correlations	Cronbach’s Alpha if item deleted
Q1	2.95	0.229	0.870
Q2	2.64	0.880	0.806
Q3A	2.62	0.886	0.805
Q3B	2.56	0.785	0.817
Q3C	2.60	0.816	0.814
Q3D	2.99	0.303	0.863
Q3E	2.98	0.305	0.863
Q3F	2.93	0.370	0.859
Q3G	2.82	0.557	0.843

Validity

**Figure 1:**
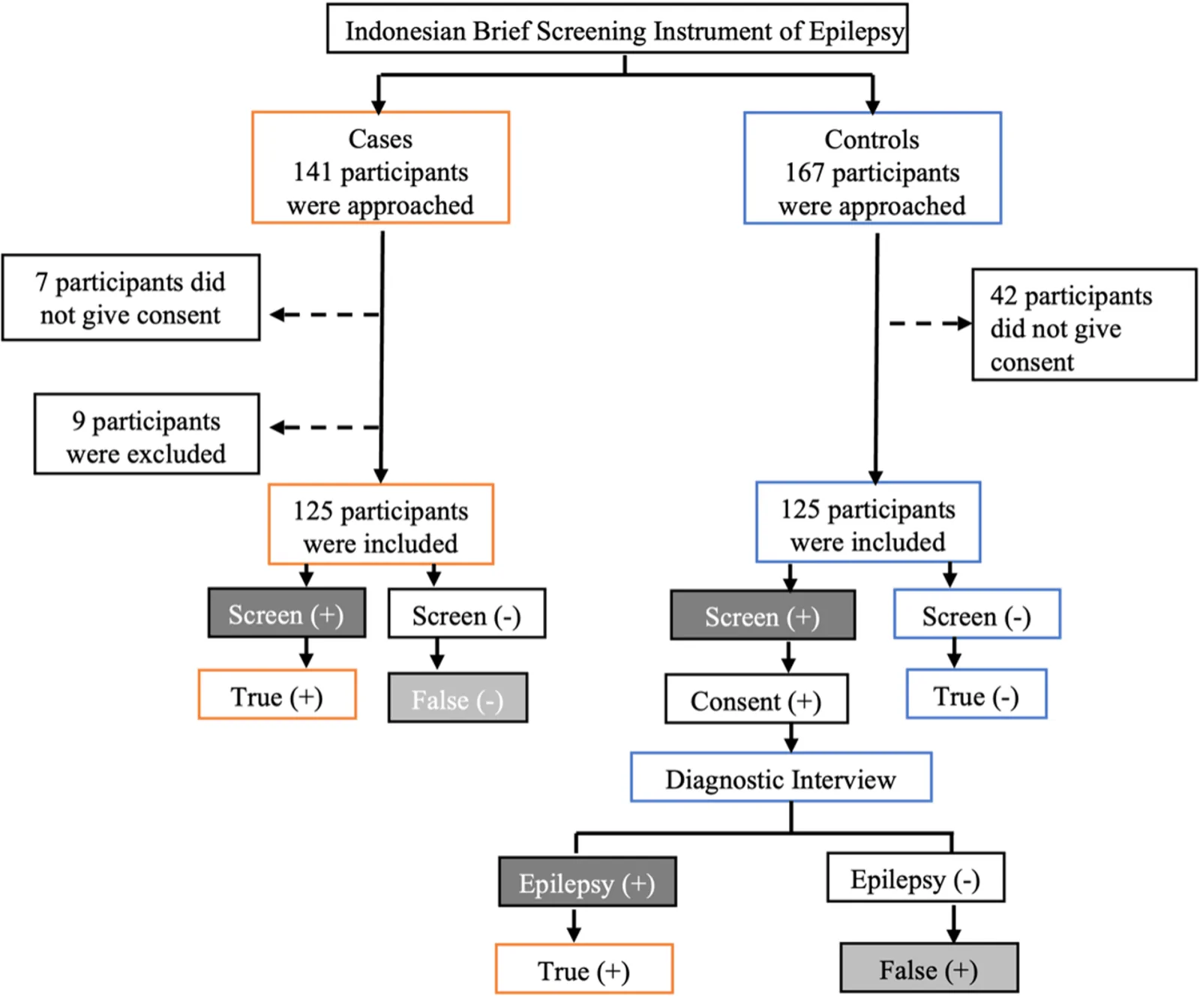
Flow diagram of subject selection

A total of 141 participants were approached for consent in the case group. Seven declined, resulting in 134 participants who were interviewed. Nine cases were excluded due to an epilepsy duration of less than one month, or the presence of cognitive impairment/mental retardation, leaving a final sample size of 125 cases. Similarly, of the 167 participants in the control group approached for consent, 42 declined, resulting in 125 controls who were interviewed, all of whom provided complete responses. Selection of participants are summarized in a flow diagram presented in Figure 1.

Among the cases, 122 (97.6%) recognized themselves as suffering from epilepsy. The remaining three answered “yes/possible” to Q3C regarding change in mental state, while two answered “yes/possible” to Q3A on experiencing any seizure. Among the controls, three subjects (2.4%) responded to a positive screen for Q2. Among the remaining 122 subjects who responded negatively to Q2, 20 of them (16.4%) answered “yes/possible” to Q3B regarding uncontrolled body movements. All responses are as stated in Table 5.

**Table 5:** Responses per item for case and controls (n=250)

Items	Responses of Cases n (%)		Responses of Controls n (%)	
	Yes/Possible	No/Don’t Know	Yes/Possible	No/Don’t Know
Q1. Febrile Seizure	35 (28.0)	90 (72.0)	12 (9.6)	113 (90.4)
Q2. Epilepsy/seizure disorder	122 (97.6)	3 (2.4)	3 (2.4)	122 (97.6)
Subjects who negatively answered Q2	n=3		n=122	
Q3A. Any seizure	2	1	2	120
Q3B. Uncontrolled body movements	2	1	20	102
Q3C. Change in mental state	3	0	10	112
Q3D. Daydreaming/staring	1	2	4	118
Q3E. Reactions to light	0	3	7	115
Q3F. Jerking/Clumsiness on awakening	1	2	8	114
Q3G. Other repeated unusual spells	1	2	6	116

Utilizing the broadest screen definition (any positive), the Indonesian version of the screening tool demonstrated a sensitivity of 100% (95% CI: 97.09-100) and a specificity of 60.8% (95% CI: 51.67-69.41), resulting in a PPV of 1.77% (95% CI: 1.42-2.19) and an NPV of 100% (95% CI: 95.26-100). When excluding positive screens for febrile seizures (Q2-Q3G), sensitivity remained at 100% (95% CI: 97.09-100), while specificity improved to 66.4% (95% CI: 59.9-76.8), increasing the PPV to 2.21% (95% CI: 1.71-2.85), with no change in NPV. When adjusting for any seizures, sensitivity slightly decreased to 99.2% (95% CI: 95.62-99.98), but specificity improved substantially to 96% (95% CI: 90.01-98.69), leading to a higher PPV of 14.88% (95% CI: 6.89-29.21) and an NPV of 99.99% (95% CI: 99.96-100). Likewise, under the strictest definition (Q2 only), sensitivity decreased to 97.6% (95% CI: 93.15-99.50), but specificity improved slightly to 97.6% (95% CI: 93.15-99.50), leading to a PPV of 22.28% (95% CI: 8.57-46.73) and an NPV of 99.98% (95% CI: 99.95-99.99). All relevant data on sensitivity, specificity, PPV, and NPV are presented in Table 6.

**Table 6:** Sensitivity, specificity, PPV, and NPV of each definition of a positive screen in comparison to the original instrument PPV: positive predictive value; NPV: negative predictive value.

Definition	History of Epilepsy (n=125)	No History of Epilepsy (n=125)	Sensitivity (95% CI)		Specificity (95% CI)		PPV (95% CI)	NPV (95% CI)
			Indonesian	Original	Indonesian	Original		
a) Any positive								
Screened (+)	125	49	100% (97.09-100)	95.8%	60.8% (51.67-69.41)	93.3%	1.77% (1.42-2.19)	100% (95.26-100)
Screened (-)	0	76						
b) Q2 (+) OR [Q2 (-) and Q3A-Q3G (+)] (excluding febrile seizure)								
Screened (+)	125	39	100% (97.09-100)	93.5%	66.4% (59.9-76.8)	94.2%	2.21% (1.71-2.85)	100% (95.80-100)
Screened (-)	0	86						
c) Q2 (+) OR [Q2 (-) and Q3A (+)] (any seizure)								
Screened (+)	124	5	99.2% (95.62-99.98)	91.1%	96.0% (90.01-98.69)	96.7%	14.88% (6.89-29.21)	99.99% (99.96-100)
Screened (-)	1	120						
d) Q2 (+) only								
Screened (+)	122	3	97.6% (93.15-99.50)	76.2%	97.6% (93.15-99.50)	99.2%	22.28% (8.57-46.73)	99.98% (99.95-99.99)
Screened (-)	3	122						

## Discussion

Demography and clinical characteristics

In our study, there were no significant differences in age or sex between the case and control groups, while marital status and educational level showed significant disparities. However, we did not adjust for these demographic imbalances, as this study focuses on cross-cultural adaptation, internal consistency, and diagnostic accuracy. Nonetheless, comprehension was equally good across both groups, likely due to the questionnaire's simple, easy-to-understand design, and the use of face-to-face guided interviews.

Reliability

The translated questionnaire demonstrated good internal consistency, with Cronbach’s alpha values of 0.857 in the pilot study (n = 30) and 0.856 in the main validation sample (n = 250). These results meet the accepted standard for reliability in screening tools. Although Item 1 had a relatively low corrected item-total correlation (-0.057 and 0.229, respectively), it was retained in the final instrument for several reasons.

First, Item 1 addresses a clinically relevant risk factor: history of febrile seizures. While not diagnostic of epilepsy, this risk factor contributes to the tool’s content validity by capturing a distinct aspect of seizure-related history. Second, retaining this item preserves consistency with the original instrument. This consistency is important to support cross-cultural comparisons and pooled analyses in future studies. In comparison, other validated translations of this instrument (French, Spanish, Malay) also retained this item. These studies, however, did not include information regarding item-total correlation of each items [5-8]. Additionally, because the questionnaire is intended for screening, maximizing sensitivity is a priority. Including Item 1 maintained 100% sensitivity in our sample, with only a modest reduction in specificity (from 66.4% to 60.8%). Given the smaller number of confirmed epilepsy cases in our sample compared to the original population-based study, retaining Item 1 may allow broader case detection, particularly in larger-scale or community-based applications. In the original validation study, the inclusion of Item 1 similarly improved sensitivity from 93.5% to 95.8% with minimal impact on specificity [5].

Validity

The translated questionnaire demonstrated high sensitivity for all the definitions of a positive screen, comparable to the original version [5]. When using the broadest screen definition (any positive response), the Indonesian version achieved a sensitivity of 100%, surpassing the original instrument's 95.8%. This indicates that our patients may have had greater awareness of their epilepsy, better knowledge of the condition, or a clearer understanding of the questionnaire items compared to respondents of the original version. However, specificity was much lower at 60%. The relatively high false-positive rate was possibly due to misinterpretation of terms like "twitching" and "changes in mental state", where participants without a history of epilepsy responded "yes/possible" to Q3B regarding "twitching" due to having experienced periodic eye twitching. The second most common cause of false-positives was Item Q3C, which addressed “changes in mental state”. This was primarily due to some participants reporting episodes of fainting, with several attributing these incidents to a history of low blood pressure. Additionally, some participants perceived Item Q3C as a form of daydreaming, which also contributed to the false-positive rates. When excluding febrile seizures (Q2-Q3G), sensitivity remained at 100%, but specificity improved. This suggests that removing febrile seizure-related items refines the screening process without compromising sensitivity. When we applied the positive definition for any seizure (Q2 or Q3A), sensitivity slightly decreased but, specificity increased significantly. This was even more pronounced when using the strictest definition (Q2 only).

This trade-off between sensitivity and specificity is a common characteristic of broad screening tools, which prioritize identifying all potential cases at the cost of increased false positives. Despite the trade-offs in specificity, the primary objective of screening instruments in large epidemiological studies is to achieve the highest possible sensitivity. Given that positive cases will undergo confirmatory diagnostic interviews, a higher sensitivity ensures that true epilepsy cases are not missed, even if it results in a lower specificity. This principle is reflected in the present findings, where the Indonesian version consistently prioritized sensitivity, making it a suitable tool for large-scale epilepsy screening.

Comparatively, other validated translations of this instrument (French, Spanish, Malay) had similar sensitivity (96.3%-100%) compared to Indonesian version when using the broadest screen definition. Specificity of the tools was also comparable, with the Malay and French version being lower, at 60.6% and 66.4%, respectively. The Spanish version, however, had a specificity of 74.77% when using the broadest screen definition. Similarly, NPV of the tools in other languages reached 100%, with PPV being low due to the low prevalence of epilepsy [5-8].

The model-derived PPV remained relatively low across all definitions as expected when applying the observed sensitivity and specificity to an assumed epilepsy prevalence of 0.7%. Conversely, the model-derived NPV approached 100%. These estimates are prevalence-dependent and were not directly observed in the case-control sample; they should therefore be interpreted cautiously as illustrative estimates of potential population-level performance rather than empirical predictive values from this cohort.

A culturally and linguistically validated screening instrument is essential for the accurate assessment of epilepsy, particularly in diverse populations such as Indonesia. Language barriers and cultural differences can significantly affect the reliability of screening tools, making it crucial to adapt and validate instruments to ensure their effectiveness in the target population. In Indonesia, where epilepsy remains underdiagnosed due to limited awareness and accessibility to healthcare services, a validated screening tool can play a critical role in early detection and intervention.

To the best of our knowledge, this is the first validated Indonesian-language epilepsy screening instrument. The adaptation of this questionnaire ensures that it is not only comprehensible but also culturally appropriate, addressing the specific ways epilepsy is perceived and described within the Indonesian community. A key strength of this study is its setting at Indonesia's national referral hospital, where the country's leading epileptologists practice and the most comprehensive facilities and resources are available. This significantly reduces the likelihood of epilepsy misdiagnosis compared to other institutions.

Limitations and future implications

This study has several limitations that should be acknowledged. The single-center design and recruitment from Indonesia’s national tertiary epilepsy referral hospital introduce selection and spectrum bias. The epilepsy group consisted of patients with established diagnosis who were receiving specialist care and may have had greater disease awareness, while controls were hospital-based patients or accompanying family members/friends. The resulting spectrum differs from that expected in community screening, where undiagnosed epilepsy, less typical presentations, and varying health literacy are more common. Consequently, the diagnostic performance observed in this study, particularly the very high sensitivity, may represent relatively optimal performance and should not assumed to apply directly to community-based populations.

Another limitation is that only screen-positive participants in the control group underwent a structured diagnostic interview, while screen-negative controls were considered true negatives without further neurological evaluation. Consequently, a small number of individuals with unrecognized epilepsy could theoretically have been misclassified as true negatives. However, given the expected low prevalence of epilepsy in the general population and the estimated NPV approaching 100%, the probability that a screen-negative participant truly had epilepsy is expected to be very small. Moreover, applying comprehensive neurological assessment to every screen-negative participant would not have been feasible in this validation study. Future population-based validation studies may strengthen the evidence by performing reference-standard evaluation in a random sample of screen-negative participants to formally assess potential verification bias.

## Conclusions

The Indonesian version of the Brief Screening Instrument for Epilepsy performed similar to the original instrument, particularly in maximizing sensitivity (100% compared to 95.8%), which is critical for a screening tool. However, the high sensitivity comes with a trade-off in specificity (60% compared to 93.3%). As such, the instrument is intended strictly for initial screening and not for diagnostic purposes, and may serve as a valid and reliable screening instrument for future population-based studies. Upcoming studies may explore the instrument's performance in different population subgroups and assess its feasibility for population-based epilepsy screening programs in Indonesia.
